# Association of left posterior pericardiotomy with postoperative atrial fibrillation in Isolated OPCAB: a propensity-weighted analysis

**DOI:** 10.1186/s13019-026-04241-3

**Published:** 2026-05-02

**Authors:** Eun Yeong Jung, Ji Eun Im, JongHyun Baek, Ho-Ki Min

**Affiliations:** 1 Department of Thoracic and Cardiovascular Surgery, Asan Medical Center, University of Ulsan College of Medicine, Seoul, Republic of Korea; 2 Department of Thoracic and Cardiovascular Surgery, Yeungnam University College of Medicine, 170 Hyeonchung-ro, Nam-gu, Daegu, 42415 Republic of Korea

**Keywords:** Left posterior pericardiotomy, Postoperative atrial fibrillation, Off-pump coronary artery bypass grafting, Pericardial effusion, Propensity score, Drainage

## Abstract

**Background:**

Off-pump coronary artery bypass grafting (OPCAB) minimizes systemic inflammation associated with cardiopulmonary bypass, yet postoperative atrial fibrillation (POAF) incidence remains substantial. This suggests that local pericardial factors may contribute significantly to POAF in this setting. We evaluated the impact of left posterior pericardiotomy (LPP) on POAF and drainage patterns in isolated OPCAB to determine if enhancing local drainage effectively mitigates POAF.

**Methods:**

We retrospectively analyzed 283 patients (mean age 65.1 ± 10.2 years; female, 18.7%) undergoing elective isolated multivessel OPCAB by a single surgeon (2021–2025). Patients were categorized into LPP (*n* = 122, routinely performed since April 2024) and no-LPP (*n* = 161) groups. Inverse probability of treatment weighting (IPTW) was applied to adjust for baseline differences. The primary endpoint was in-hospital POAF. Postoperative chest tube drainage distribution and incidence of postoperative pericardial effusion were analyzed to assess the mechanistic efficacy of LPP.

**Results:**

After IPTW adjustment, the LPP group showed a significantly lower incidence of POAF (15.1% vs. 30.7%; *p* = 0.003) and shorter hospital length of stay (*p* = 0.032). Operative time was significantly shorter in the LPP group (334 ± 44 vs. 349 ± 48 min; *p* = 0.006), suggesting that LPP did not prolong the procedure. Operative mortality and LPP-related complications were absent. Postoperative pericardial effusion was also less frequent in the LPP group (3.2% vs. 8.4%; *p* = 0.084). Mechanistically, the LPP group demonstrated a significantly higher left-to-total drainage ratio in the early postoperative period, indicating effective diversion of pericardial fluid to the left pleural space during the critical early postoperative period.

**Conclusions:**

In elective isolated OPCAB, concomitant LPP was associated with a lower incidence of POAF and shorter hospital length of stay without increasing operative time or complications. The observed shift in drainage distribution supports the mechanism that LPP reduces POAF by effectively mitigating local pericardial fluid retention.

**Supplementary Information:**

The online version contains supplementary material available at 10.1186/s13019-026-04241-3.

## Background

Postoperative atrial fibrillation (POAF) is among the most common complications after cardiac surgery, with a reported incidence of approximately 30% to 50% depending on the type of operation and the rhythm surveillance strategy [1–3]. POAF has been associated with adverse outcomes, including stroke, increased mortality, prolonged hospitalization, and higher healthcare utilization [1, 2]. Despite its clinical impact, preventive strategies have largely relied on pharmacologic prophylaxis and treatment (e.g., beta-blockers, amiodarone, magnesium), whereas intraoperative surgical techniques remain less consistently adopted in routine practice [3].

Left posterior pericardiotomy (LPP), a simple incision connecting the pericardial space to the left pleural cavity, has been proposed as a surgical adjunct to reduce POAF by facilitating continuous pericardial drainage and limiting effusion-related mechanical and inflammatory stimuli to the atria [4–8]. Randomized trials and meta-analyses have supported its efficacy in broader cardiac surgery populations, and recent guidelines have begun to incorporate posterior pericardiotomy as a preventive strategy for POAF [3–5]. In addition, atrial fibrillation burden and postoperative recurrence after cardiac surgery have been associated with worse long-term outcomes, underscoring the clinical importance of perioperative AF prevention and management strategies [9, 10]. Nevertheless, adoption of LPP remains variable, and evidence gaps persist across specific operative contexts.

Although the rationale for LPP is not inherently dependent on cardiopulmonary bypass (CPB), most evidence derives from mixed cardiac surgery cohorts or CABG populations in which CPB is commonly used [4–8]. POAF pathophysiology involves both systemic and local inflammatory pathways [11]. While off-pump coronary artery bypass grafting (OPCAB) avoids CPB-induced systemic inflammation [12–14], the incidence of POAF remains substantial in this setting [2, 15]. This discrepancy suggests that local pericardial factors may be the dominant driver of POAF in the absence of CPB. Accordingly, specific data are needed to determine whether the reported benefits of LPP generalize to an isolated OPCAB population.

At our institution, LPP has been routinely performed during OPCAB since April 2024 unless contraindicated, enabling comparison with a historical cohort of patients who underwent isolated OPCAB without LPP prior to this practice change. We designed this study to evaluate the association of concomitant LPP with POAF and related early postoperative outcomes in an isolated OPCAB cohort.

## Methods

### Study design and patient population

This retrospective, single-center, single-surgeon cohort study included consecutive adult patients who underwent OPCAB at our institution between January 2021 and October 2025. Eligible patients were adults who underwent elective, isolated OPCAB for multivessel coronary artery disease involving at least two target vessels, via full median sternotomy. Since April 2024, LPP has been routinely performed during OPCAB at our institution unless contraindicated. Patients were categorized into two groups according to whether concomitant LPP was performed or not. The following cases were excluded: (1) Use of cardiopulmonary bypass (including on-pump beating heart CABG or conventional on-pump CABG), or intraoperative conversion from OPCAB to on-pump CABG; (2) Cases in which LPP was not feasible or not performed because of severe lung disease, pleural adhesions or any other reasons; (3) Single-vessel disease or minimally invasive direct coronary artery bypass; (4) History of chronic AF or other sustained atrial arrhythmias, regardless of medication status (patients with new-onset AF related to the current angina episode were included in this study); (5) Emergency or Urgent surgery; (6) Concomitant cardiac procedures in addition to CABG; (7) Prior cardiothoracic surgery; (8) Cases in which the left pleural space was not opened and a left pleural drain was not placed, to ensure comparable postoperative drainage management.

### Study outcomes

The primary outcome was the incidence of POAF during the index hospitalization. Additional perioperative and postoperative outcomes included in-hospital mortality, total operative time, intensive care unit (ICU) length of stay, hospital length of stay, reoperation for postoperative bleeding, low-cardiac-output syndrome (LCOS) requiring mechanical circulatory support, and serial chest tube drainage volumes over time, analyzed separately for the left and right chest tubes.

### Surgical procedure

**Fig. 1:**
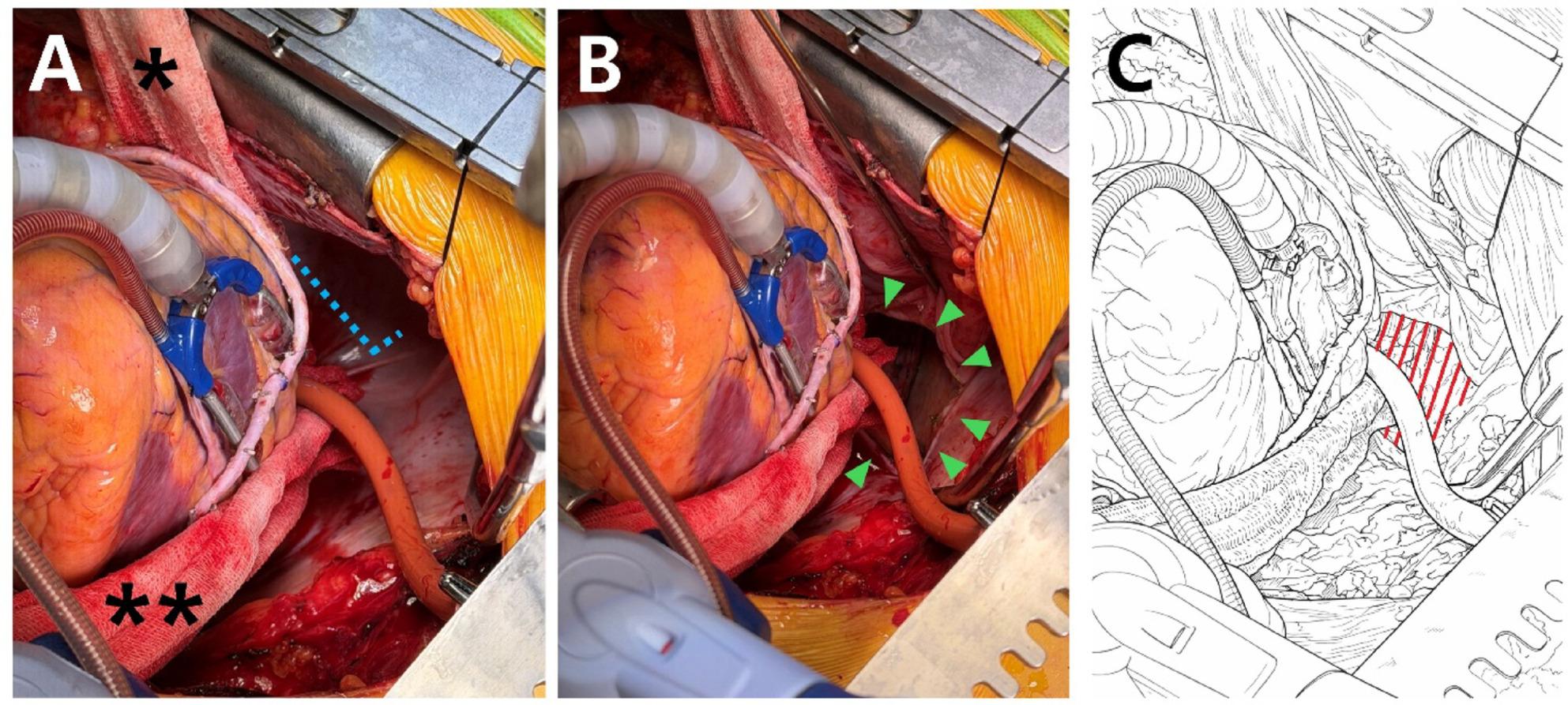
Exposure and incision for left posterior pericardiotomy. Representative views of the left posterior pericardium before (**A**) and after left posterior pericardiotomy (**B**, **C**) . A long fabric band is anchored at its midpoint to the posterior pericardium between the left inferior pulmonary vein and the inferior vena cava (sling technique). The left arm of the band (*) is retracted cephalad and the right arm (**) is pulled rightward to expose the left posterior pericardium (**A**). A reversed L-shaped pericardial incision (blue dotted line) is then made parallel to the phrenic nerve and extended approximately 1 cm anteriorly (**B**). The opened margins are indicated by green arrowheads (**B**), and the pericardial opening is shown by red hatching (**C**)

**Fig. 2:**
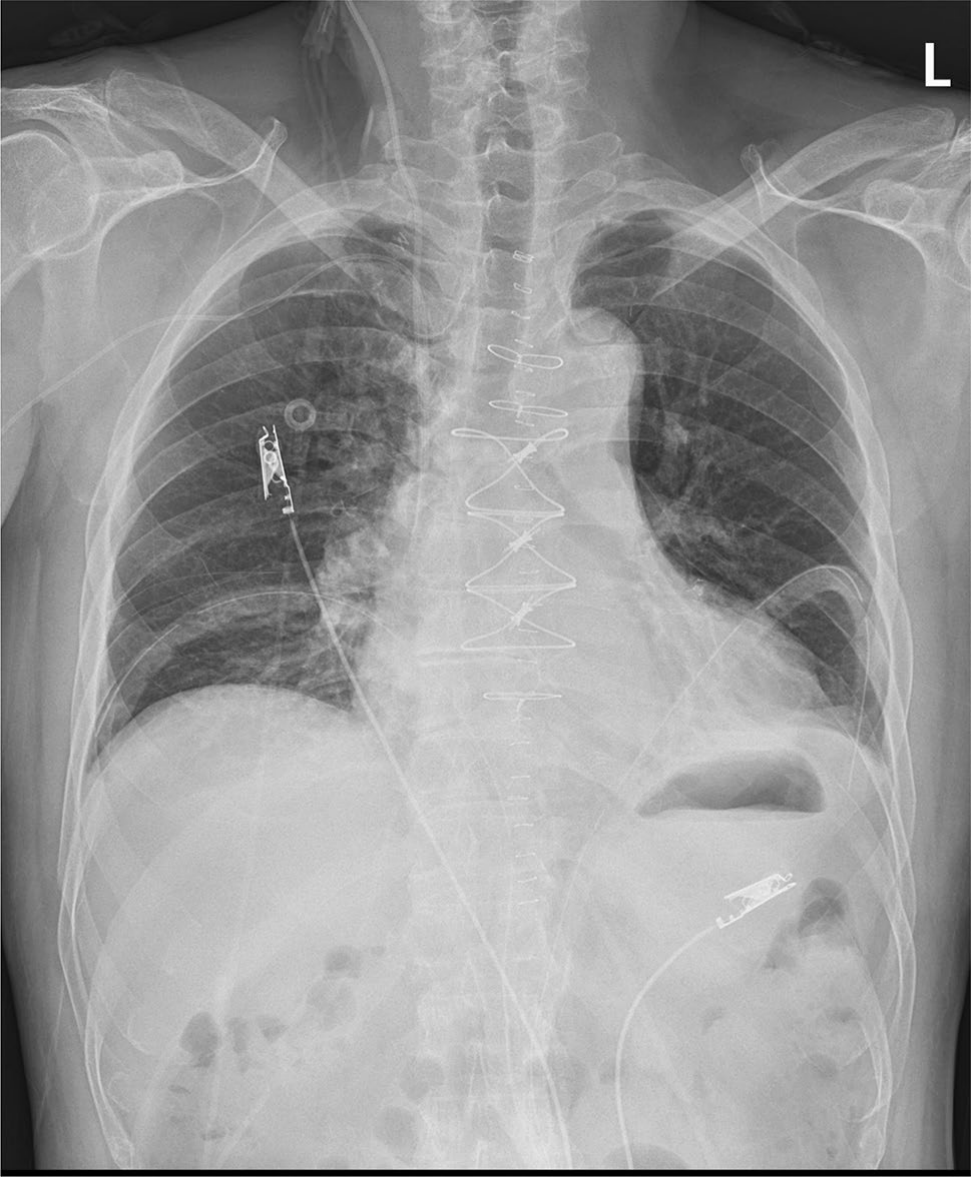
Two-pleural chest tube placement after OPCAB. Postoperative chest radiograph showing two chest tubes, one in each pleural space, with both tips directed toward the diaphragm.

Anesthetic management and surgical techniques were similar in both groups. Patients were positioned supine, and a full median sternotomy was performed. In almost all patients, an in situ left internal mammary artery to left anterior descending artery anastomosis was routinely performed. As a second conduit, the right internal mammary artery (RIMA) was preferentially used according to our institutional strategy; when RIMA use was not feasible, an alternative conduit was selected on a case-by-case basis. Distal anastomoses were usually performed using a composite graft strategy that avoided aortic manipulation. The sling technique was applied to facilitate exposure of the lateral or posterior wall (Fig. 1A). Generally, LPP was performed after completion of the lateral wall anastomosis to minimize the risk of graft injury associated with LPP. LPP consisted of a 6-cm longitudinal incision made parallel and posterior to the left phrenic nerve, extending from the left inferior pulmonary vein to the diaphragm. Once the incision reached the diaphragm, it was extended approximately 1 cm anteriorly to create a reverse “L”-shaped incision to facilitate drainage (Fig. 1A, blue dot; Fig. 1B, green arrow). In all patients, only two chest tubes were used, and no additional drains were inserted. A chest tube was inserted into each pleural space; the tubes were not inserted through the LPP incision but were instead placed through the pleural slit. Each tube was positioned with its tip directed toward the diaphragm (Fig. 2). In both groups, no drain was placed directly in the posterior pericardial space to avoid graft injury and reduce mechanical irritation of the heart. The pericardium was left open.

### Postoperative care, AF surveillance, and definition of POAF

All patients in both cohorts received standardized postoperative care, including continuous electrocardiogram (ECG) monitoring throughout hospitalization. β-blockers were initiated from postoperative day 1 in hemodynamically stable patients (mean arterial pressure ≥ 60 mmHg and heart rate ≥ 60 beats per minute). When AF was detected and/or sinus rhythm was restored, a 12-lead ECG was obtained for documentation. During postoperative recovery, patients underwent daily laboratory evaluations, including electrolytes and cardiac enzymes, and routine monitoring of vital signs and urine output.

POAF was defined as new-onset atrial fibrillation or atrial flutter characterized by the absence of discernible P waves and lasting ≥ 5 min during the index hospitalization. To reduce misclassification due to transient postoperative factors, reversible contributors (electrolyte or volume abnormalities) were corrected first when atrial arrhythmia was detected. Episodes that resolved within 30 min after correction of these reversible factors, without additional pharmacologic or electrical intervention, were not considered POAF events. Episodes requiring pharmacologic or electrical intervention were classified as POAF regardless of duration, consistent with previously published definitions [3, 4]. Inotropic agents potentially contributing to atrial arrhythmogenesis were tapered or discontinued as early as clinically feasible based on hemodynamic stability. For patients meeting POAF criteria, amiodarone was administered intravenously and transitioned to oral therapy after rhythm conversion; electrical cardioversion was performed when clinically indicated.

### Chest drainage management and imaging follow-up

Chest tube drains were connected to a standard negative-pressure suction system (-15 to -20 cmH2O), and tube patency was routinely verified. Drainage volume was recorded hourly on the operative day and at least once daily according to institutional protocol. Chest tubes were removed when drainage decreased to < 100 mL over 24 h on any postoperative day. Before discharge, two-dimensional transthoracic echocardiography and three-dimensional cardiac computed tomography (CT) were routinely performed after removal of both chest tubes to evaluate pericardial effusion and graft patency. CT was not performed in patients with renal insufficiency.

### Statistical analysis

All statistical analyses were performed using R (R Foundation for Statistical Computing, Vienna, Austria). Continuous variables are presented as mean ± standard deviation or median (interquartile range), as appropriate, and categorical variables as counts and percentages. Baseline characteristics between groups were compared in the unmatched cohort using the Student t test or Wilcoxon rank-sum test for continuous variables and the χ² test or Fisher exact test for categorical variables.

To reduce confounding related to treatment selection, propensity scores for undergoing LPP were estimated using multivariable logistic regression including clinically relevant preoperative covariates. Inverse probability of treatment weighting (IPTW) was then applied using stabilized weights to estimate the average treatment effect. Covariate balance before and after weighting was assessed using standardized mean differences (SMDs), with an absolute SMD < 0.20 considered acceptable and an absolute SMD < 0.10 considered indicative of excellent balance. Comparative analyses in the weighted cohort were performed using weighted regression models according to outcome type, with weighted logistic regression for binary outcomes and weighted linear regression for continuous outcomes, and results were reported using two-sided p values. Additionally, logistic regression analyses were performed to evaluate the association between operative time and POAF. Operative time was modeled per 10-minute increase. Both unadjusted and multivariable models were fitted, and results are reported as odds ratios (OR) with 95% confidence intervals (CI). A two-sided p value < 0.05 was considered statistically significant.

### Declaration of generative AI and AI-assisted technologies in the manuscript preparation process

During the preparation of this work, the author(s) used ChatGPT (OpenAI) to assist with English-language editing and statistical consultation (e.g., clarifying analytical approaches and reporting). After using this tool, the author(s) reviewed and edited the content as needed and take full responsibility for the content of the published article.

### Ethical approval

The study protocol was reviewed and approved by the Institutional Review Board of Yeungnam University Medical Center (Approval Number: 2026-01-006). The requirement for informed consent was waived owing to the retrospective nature of the study.

## Results

Between January 2021 and October 2025, 452 consecutive patients underwent CABG at our institution. After applying the prespecified exclusion criteria, 283 patients who underwent elective isolated OPCAB were included in the final cohort (LPP, n = 122; no-LPP, n = 161) (Figure E1). Baseline characteristics are summarized in Table 1. Before weighting, the LPP group was older (66.49 ± 10.06 vs. 63.98 ± 10.27 years; p = 0.032), had a lower BMI (22.45 ± 3.38 vs. 24.13 ± 3.46; p < 0.001), and had a higher prevalence of diabetes mellitus (64.8% vs. 50.3%; p = 0.016). The proportion of female patients was also higher in the LPP group (24.6% vs. 14.3%; p = 0.032). The LPP group underwent fewer coronary anastomoses (3.80 ± 0.90 vs. 4.07 ± 0.89; p = 0.016). After IPTW using stabilized weights, baseline covariates were well balanced, with improved covariate balance across all variables (Table 1; Figure E2).Operative time was significantly shorter in the LPP group in the unmatched cohort (328.68 ± 44.26 vs 355.53 ± 47.63 minutes; p < 0.001). After IPTW adjustment, operative time remained significantly shorter in the LPP group (333.61 ± 43.79 vs 348.97 ± 47.67 minutes; p = 0.006) (Table 2). 

### Postoperative outcomes

The primary outcome, POAF, occurred significantly less frequently in the LPP group in the unmatched cohort (18.0% vs. 31.7%; p = 0.009). This association persisted after IPTW adjustment (15.1% vs. 30.7%; p = 0.003) (Table 2). Postoperative pericardial effusion was also lower in the LPP group before weighting (1.6% vs. 8.1%; p = 0.017) and remained numerically lower after IPTW adjustment (3.2% vs. 8.4%; p = 0.084). Early mortality was 0.0% in the LPP group and 5.0% in the no-LPP group in the unmatched cohort. After IPTW adjustment, early mortality remained 0.0% in the LPP group and 4.7% in the no-LPP group. ICU stay (hours) did not differ between groups before or after IPTW adjustment (p = 0.785 and p = 0.671, respectively). In contrast, hospital length of stay (day) was shorter in the LPP group both before and after IPTW adjustment (p = 0.033 and p = 0.032, respectively). Postoperative bleeding requiring surgical re-intervention and mechanical circulatory support were rare and did not differ significantly after IPTW adjustment (Table 2). 

### Drain output and drainage distribution

**Fig. 3:**
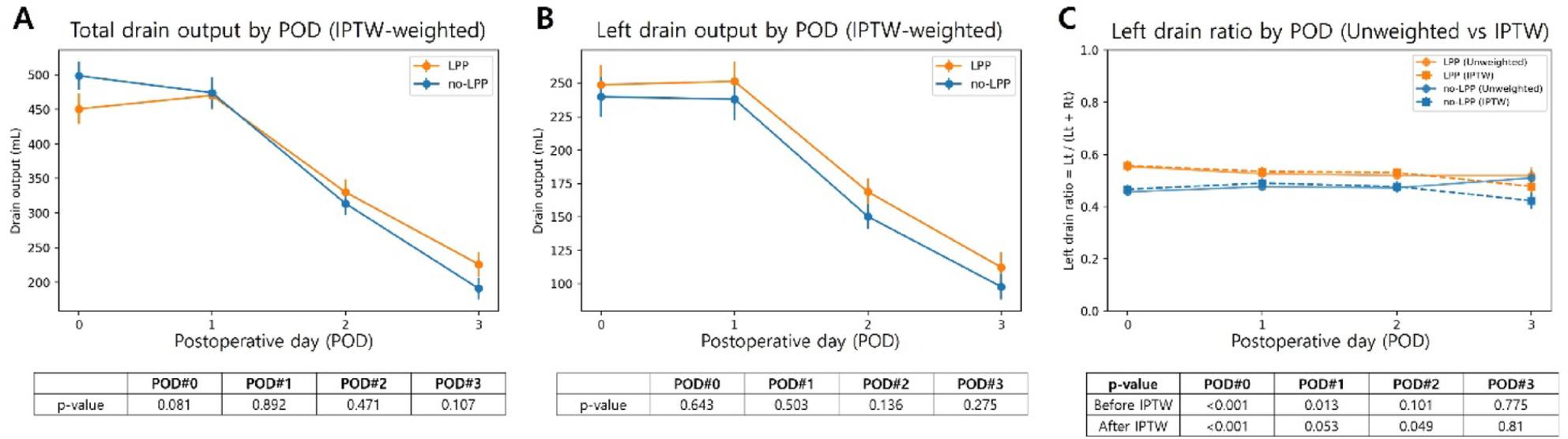
Postoperative drainage after OPCAB with and without LPP. IPTW-weighted total drain output by POD is shown in (**A**), and IPTW-weighted left drain output by POD is shown in (**B**). The left drain ratio (Left / [Left + Right]) by POD in the unweighted cohort and after IPTW adjustment is shown in (**C**). p-values for between group comparisons at each POD are shown beneath each panel. POD, postoperative day; IPTW, Inverse probability treatment weighting; LPP, Left Posterior pericardiotomy

Figure 3 shows postoperative drain output trends. Total and left-sided drain outputs decreased over postoperative days in both groups, with broadly similar trajectories (Figure 3A, 3B). To assess drainage distribution, we analyzed the left drain ratio, defined as Left / (Left + Right) (Figure 3C). In the unadjusted cohort, the left drain ratio differed between groups on POD#0 (p < 0.001) and POD#1 (p = 0.013) but not on POD#2 or POD#3. After IPTW adjustment, the between-group difference remained significant on POD#0 (p < 0.001) and became significant on POD#2 (p = 0.049), whereas differences were not significant on POD#1 (p = 0.053) or POD#3 (p = 0.81). Overall, the left drain ratio remained consistently higher in the LPP group throughout the observation period.

## Discussion

In this IPTW-adjusted analysis of elective isolated OPCAB, concomitant LPP was associated with a lower incidence of POAF and a shorter hospital length of stay, without an apparent increase in early adverse outcomes. These findings extend prior evidence supporting posterior pericardiotomy to an OPCAB-specific cohort, in which data have been limited [4–8].

Small-to-moderate pericardial effusions are frequently observed after cardiac surgery and have been implicated in the pathogenesis of POAF [16]. LPP creates a continuous drainage pathway between the pericardial and left pleural spaces, facilitating evacuation of pericardial fluid during the early postoperative period. One plausible mechanism is that even small amounts of pericardial fluid adjacent to the atria may promote atrial arrhythmogenesis through mechanical compression and local inflammatory and oxidative pathways [4, 7, 16]. Continuous pericardial drainage via LPP may mitigate these arrhythmogenic substrates. Consistent with this hypothesis, our drainage analysis demonstrated a greater left-sided contribution to chest tube output during early postoperative period in the LPP group, reflected by an increased left-to-total drainage ratio. Notably, after IPTW adjustment, total drainage volume did not differ between groups, supporting a redistribution of drainage toward the left side rather than an overall increase in drainage volume (Fig. 3). This direction-specific shift is consistent with preferential diversion of pericardial fluid into the left pleural space during the early postoperative period, when POAF most commonly develops, and aligns with prior mechanistic work linking pericardial effusion to postoperative atrial arrhythmogenesis [4, 7, 16].

In addition, because postoperative atrial arrhythmogenesis may be promoted by local pericardial inflammation and retained blood adjacent to the atria, a pericardial opening does not necessarily need to be created in close proximity to the atrial free wall; instead, placing the incision at a dependent location to facilitate drainage may be preferable. Rather, an opening placed too close to the atrial free wall could theoretically increase mechanical irritation during the early postoperative period. Accordingly, LPP was created at the dependent portion of pericardium to promote effective drainage into the left pleural space while minimizing direct atrial manipulation [17].

Prior randomized trials and meta-analyses have shown that posterior pericardiotomy reduces POAF across broader cardiac surgery populations [4, 5]. Because this drainage-based rationale is not inherently dependent on CPB, its physiologic basis would be expected to apply in both on-pump and off-pump CABG. In this isolated OPCAB cohort, concomitant LPP was associated with a lower incidence of POAF after IPTW adjustment, extending prior evidence to an OPCAB-specific setting where data remain limited. We observed no significant difference in LPP-attributable complications, and hospital length of stay was shorter in the LPP group both before and after IPTW adjustment. Collectively, these findings suggest that LPP may represent a simple and effective adjunctive maneuver to reduce POAF after OPCAB without an apparent increase in perioperative morbidity.

From a technical standpoint, we aimed to facilitate postoperative drainage while minimizing the risk of graft or anastomotic injury and supporting early mobilization. Specifically, we used only two chest tubes in all patients, without additional drains. A chest tube was inserted into each pleural space through the pleural slit (rather than through the LPP incision), and each tube was positioned with its tip directed toward the diaphragm (Fig. 2). This standardized approach may provide effective postoperative drainage while avoiding direct manipulation near coronary conduits. Postoperative pericardial effusion assessed by routine postoperative echocardiography or CT was also less frequent in the LPP group, supporting the successful drainage of pericardial effusions by our strategy.

Specifically, PE was significantly less frequent in the LPP group than in the no-LPP group (1.6% vs. 8.1%; *p* = 0.02; Table 2). PE was numerically more common among patients who developed POAF (8.0% vs. 4.3%), but this association did not reach statistical significance (*p* = 0.24), and effusion grade (small vs. moderate) was not associated with POAF occurrence (*p* = 0.60). These analyses were limited by the low PE event count (*n* = 15), and larger studies with standardized effusion grading are warranted to clarify the relationship between effusion severity and postoperative arrhythmogenesis (Supplementary table E1).

All 8 early mortality cases occurred in the no-LPP group, of whom 4 had developed POAF before death. A sensitivity analysis excluding these patients (*n* = 275) yielded consistent results (POAF: 18.0% vs. 31.0%; *p* = 0.025), indicating that the observed association was not attributable to competing risk from early mortality.

Operative time was shorter in the LPP group in both the unadjusted and IPTW-adjusted analyses; however, operative time itself was not associated with POAF in exploratory logistic regression analyses (per 10-minute increase: unadjusted OR 0.98, 95% CI 0.92–1.03; *p* = 0.43; adjusted OR 0.99, 95% CI 0.92–1.07; *p* = 0.79).

### Study limitations

Several limitations should be considered. First, this was a retrospective, single-center study performed by a single surgeon. Although we applied IPTW using clinically relevant covariates to reduce confounding, residual confounding cannot be excluded, and external validity may be limited. Second, the comparison involved patients treated during different time periods, including a pre–April 2024 period before routine implementation of LPP, introducing the potential for temporal bias related to changes in perioperative management, institutional practice patterns, or cumulative surgical experience over time. Third, to reduce heterogeneity, patients undergoing concomitant valve procedures and those with a history of atrial arrhythmias were excluded; therefore, the applicability of these findings to broader cardiac surgical populations remains uncertain. Fourth, continuous rhythm monitoring during hospitalization enabled detection of arrhythmic episodes including subclinical events; the magnitude of the observed association may differ if POAF were restricted to clinically overt or treatment-requiring episodes. Fifth, although most patients who developed POAF received treatment, detailed arrhythmia burden (e.g., total duration) was not available. Finally, the sample size limited statistical power for subgroup analyses, and prospective multicenter studies are needed to confirm these findings and clarify longer-term clinical implications.

## Conclusion

Concomitant LPP during elective isolated OPCAB was associated with a lower incidence of POAF without procedure-related complications or an apparent increase in perioperative resource utilization. Postoperative drainage patterns showed a greater left-sided contribution to total chest tube output in the LPP group during the early postoperative period, supporting the concept that enhanced pericardial-to-pleural drainage may contribute to the reduction in POAF. Given its simplicity, safety, and potential efficacy, LPP may serve as a valuable adjunct for POAF prevention in OPCAB.

**Table 1:** Baseline characteristics of OPCAB patients with and without LPP

Characteristics	Total (*N* = 283)	Before Matching				After IPTW			
		LPP (*N* = 122)	No-LPP (*N* = 161)	*p*-value	SMD	LPP (*N* = 122)	No-LPP (*N* = 161)	*p*-value	SMD
Age	65.06 ± 10.24	66.49 ± 10.06	63.98 ± 10.27	0.03	0.25	63.87 ± 11.64	65.04 ± 10.24	0.37	0.11
Female	53 (18.7%)	30 (24.6%)	23 (14.3%)	0.03	0.26	23 (18.3%)	29 (18.0%)	0.94	0.009
BMI	23.41 ± 3.52	22.45 ± 3.38	24.13 ± 3.46	< 0.001	0.49	23.65 ± 3.83	23.37 ± 3.65	0.53	0.08
Previous PCI	62 (21.9%)	25 (20.5%)	37 (23.0%)	0.67	0.06	27 (21.8%)	35 (21.9%)	> 0.99	0.001
Recent MI^a)^	155 (54.8%)	70 (57.4%)	85 (52.8%)	0.47	0.09	69 (55.7%)	88 (54.7%)	0.87	0.02
3vs dz.^b)^	227 (80.2%)	101 (82.8%)	126 (78.3%)	0.37	0.11	100 (81.1%)	129 (80.8%)	0.96	0.006
DM	160 (56.5%)	79 (64.8%)	81 (50.3%)	0.02	0.29	69 (55.5%)	87 (54.6%)	0.88	0.02
HTN	170 (60.1%)	68 (55.7%)	102 (63.4%)	0.22	0.16	72 (58.4%)	94 (58.9%)	0.93	0.01
ESRD	41(14.5%)	14 (11.5%)	27 (16.8%)	0.24	0.15	20 (16.0%)	25 (15.4%)	0.88	0.02
LVEF < 55%	142 (50.2%)	57 (46.7%)	85 (52.8%)	0.34	0.12	58 (46.8%)	78 (48.7%)	0.76	0.04
STS score									
Low risk^c)^	198 (70.0%)	82 (67.2%)	116 (72.0%)	0.43	0.11	82 (66.7%)	111 (69.4%)	0.63	0.06
Intermediate risk^d)^	64(22.6%)	34 (27.9%)	30 (18.6%)	0.09	0.22	32 (26.3%)	38 (23.9%)	0.65	0.06
High risk^e)^	21 (7.4%)	6 (4.9%)	15 (9.3%)	0.18	0.17	9 (7.0%)	11 (6.7%)	0.91	0.01
# of anastomosis^f)^	3.95 ± 0.90	3.80 ± 0.90	4.07 ± 0.89	0.02	0.31	3.94 ± 0.96	3.95 ± 0.88	0.96	0.006

Values are presented as n (%) or mean (standard-deviation)

OPCAB, off-pump coronary artery bypass grafting; LPP, left posterior pericardiotomy; IPTW, inverse probability of treatment weighting; SMD, standardized mean difference; BMI, body mass index; PCI, percutaneous coronary intervention; MI, myocardial infarction; 3vs dz., 3 vessel disease; DM, diabetes mellitus; HTN, hypertension; ESRD, end-stage renal disease; LVEF, left ventricular ejection fraction; STS, Society of Thoracic Surgeons

a) Elective surgery performed in patients with acute myocardial infarction within 1 week prior to operation

b) Patients diagnosed with 3-vessel disease who underwent ≥ 3 coronary anastomoses during surgery

c) STS score < 2

d) STS score 2–5

e) STS score > 5

f) Number of coronary artery anastomosis

**Table 2:** Perioperative outcomes before and after IPTW in OPCAB patients

Variables	Total (*N* = 283)	Before matching			After IPTW		
		LPP (*N* = 122)	No-LPP (*N* = 161)	*p*-value	LPP (*N* = 122)	No-LPP (*N* = 161)	*p*-value
Intraoperative							
OP time	343.95 ± 48.01	328.68 ± 44.26	355.53 ± 47.63	< 0.001	333.61 ± 43.79	348.97 ± 47.67	0.006
Postoperative							
Postop AF	73 (25.8%)	22 (18.0%)	51 (31.7%)	0.009	19 (15.1%)	49 (30.7%)	0.003
Postop PE^a)^	15 (5.3%)	2 (1.6%)	13 (8.1%)	0.02	4 (3.2%)	13 (8.4%)	0.08
Early mortality^b)^	8 (2.8%)	0 (0.0%)	8 (5.0%)	0.01	0 (0.0%)	8 (4.7%)	> 0.99
ICU stay (hours)	46.53 ± 32.55	47.08 ± 16.19	46.12 ± 40.85	0.79	47.42 ± 15.80	45.84 ± 38.58	0.67
Hospital stays (day)	14.53 ± 11.77	12.98 ± 6.00	15.71 ± 14.61	0.03	12.85 ± 6.04	15.86 ± 14.47	0.03
Postop bleeding^b)^	1 (0.4%)	1 (0.8%)	0 (0.0%)	0.43	1 (0.9%)	0 (0.0%)	> 0.99
Postop MCS	6 (2.1%)	2 (1.6%)	4 (2.5%)	0.7	1 (1.1%)	4 (2.2%)	0.49

Values are presented as n (%) or mean (standard-deviation)

IPTW, inverse probability of treatment weighting; OPCAB, off-pump coronary artery bypass grafting; LPP, left posterior pericardiotomy; OP time, operation time; AF, atrial fibrillation; PE, pericardial effusion, ICU, intensive care unit; MCS, mechanical circulatory support

a) Postoperative pericardial effusion was assessed using routine postoperative echocardiography or computed tomography

b) Early mortality was defined as any death occurring within 30 days after surgery or during the index hospitalization before discharge

c) Bleeding requiring surgical re-intervention

## Electronic Supplementary Material

Below is the link to the electronic supplementary material.

Additional file 1: Figure E1. Study flow diagram. This diagram shows patient selection, exclusion criteria, and final cohort allocation to the LPP and no-LPP groups for analysis. Abbreviations: CABG, coronary artery bypass grafting; Hx., History; AF, atrial fibrillation; CPB, cardiopulmonary bypass; LPP, left posterior pericardiotomy; IPTW, inverse probability of treatment weighting

Additional file 2: Figure E2. Covariate balance before and after IPTW (love plot). Love plot showing absolute standardized mean differences before (blue) and after (orange) inverse probability of treatment weighting for the LPP and no-LPP groups. Vertical reference lines indicate thresholds for excellent (0.10) and acceptable (0.20) balance. Abbreviations: BMI, body mass index; PCI, percutaneous coronary intervention; MI, myocardial infarction; 3vs dz., 3 vessel coronary artery disease; DM, diabetes mellitus; HTN, hypertension; ESRD, end-stage renal disease; LVEF, left ventricular ejection fraction; STS score, Society of Thoracic Surgeons score. a) History of previous percutaneous coronary intervention. b) Patients with acute myocardial infarction within 1 week prior to operation. c) Number of coronary anastomoses.

Additional file 3: Table E1. Characteristics of postoperative pericardial effusion in patients with and without left posterior pericardiotomy. Effusion size was graded according to the 2025 ACC concise guidance (small <1.0 cm, moderate 1.0–1.9 cm, large 2.0–2.5 cm, very large >2.5 cm). Of 283 patients, 15 (5.3%) had detectable PE; 13 in the no-LPP group and 2 in the LPP group. Abbreviations: LPP, left posterior pericardiotomy; POAF, postoperative atrial fibrillation

## Data Availability

The datasets generated and/or analysed during the current study are not publicly available due to privacy and ethical restrictions regarding patient information, but are available from the corresponding author on reasonable request.

## Abbreviations

POAF: Postoperative atrial fibrillation
AF: Atrial fibrillation
LPP: Left posterior pericardiotomy
OPCAB: Off-pump coronary artery bypass grafting
CABG: Coronary artery bypass grafting
ICU: Intensive care unit
LCOS: Low cardiac output syndrome
LIMA: Left internal mammary artery
LAD: Left anterior descending artery
RIMA: Right internal mammary artery
ECG: Electrocardiogram
TTE: Transthoracic echocardiography
CT: Computed tomography
IPTW: Inverse probability treatment weighting
SMD: Standardized mean difference
