# Supplementary figures and images for: Association of left posterior pericardiotomy with postoperative atrial fibrillation in Isolated OPCAB: a propensity-weighted analysis
Source: J Cardiothorac Surg. 2026 May 2;21:437. doi: 10.1186/s13019-026-04241-3 (PMC13285440; doi:10.1186/s13019-026-04241-3)

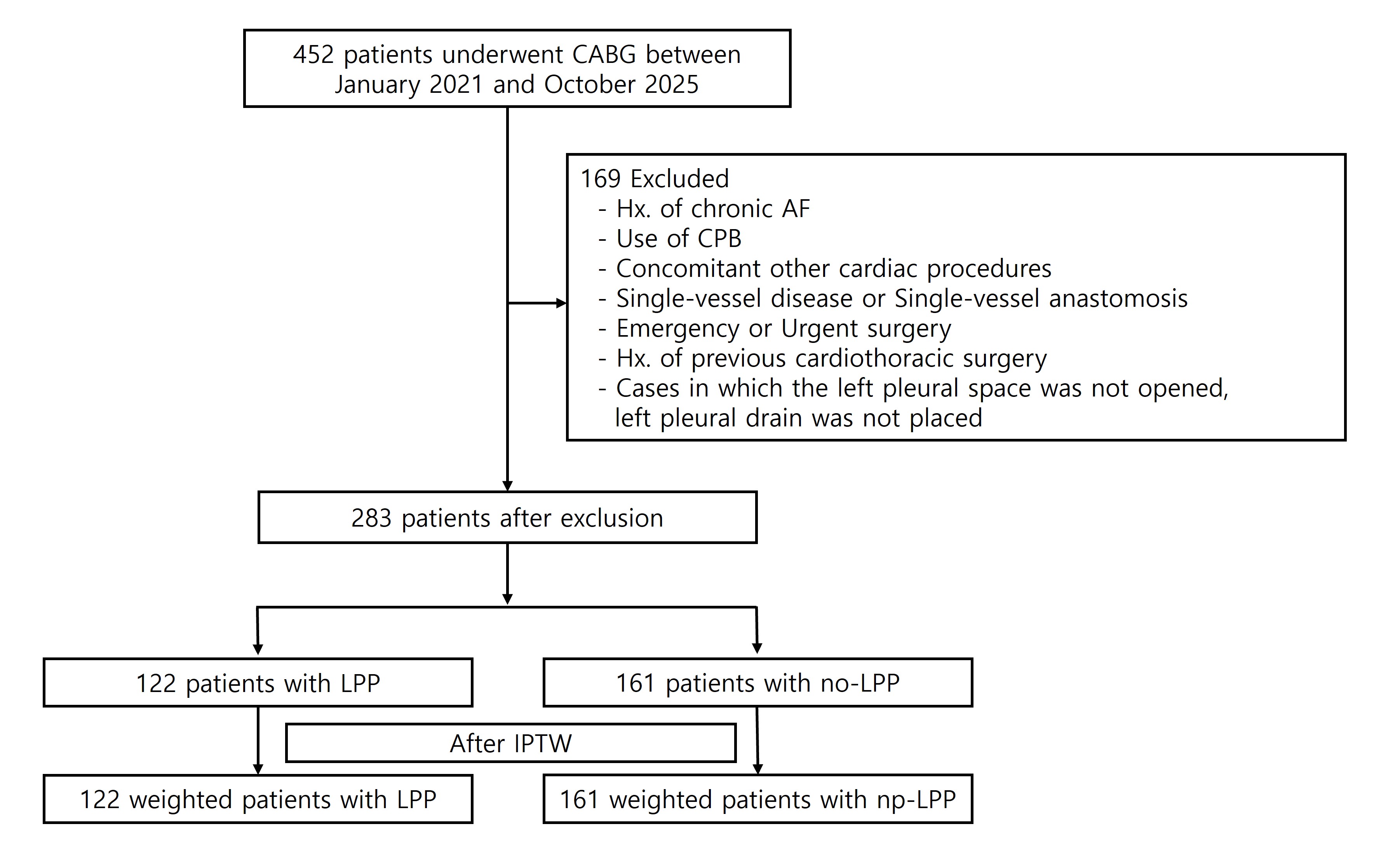

Supplement: Supplementary file 1 — Additional file 1: Figure E1. Study flow diagram. This diagram shows patient selection, exclusion criteria, and final cohort allocation to the LPP and no-LPP groups for analysis. Abbreviations: CABG, coronary artery bypass grafting; Hx., History; AF, atrial fibrillation; CPB, cardiopulmonary bypass; LPP, left posterior pericardiotomy; IPTW, inverse probability of treatment weighting [file 13019_2026_4241_MOESM1_ESM.jpg]

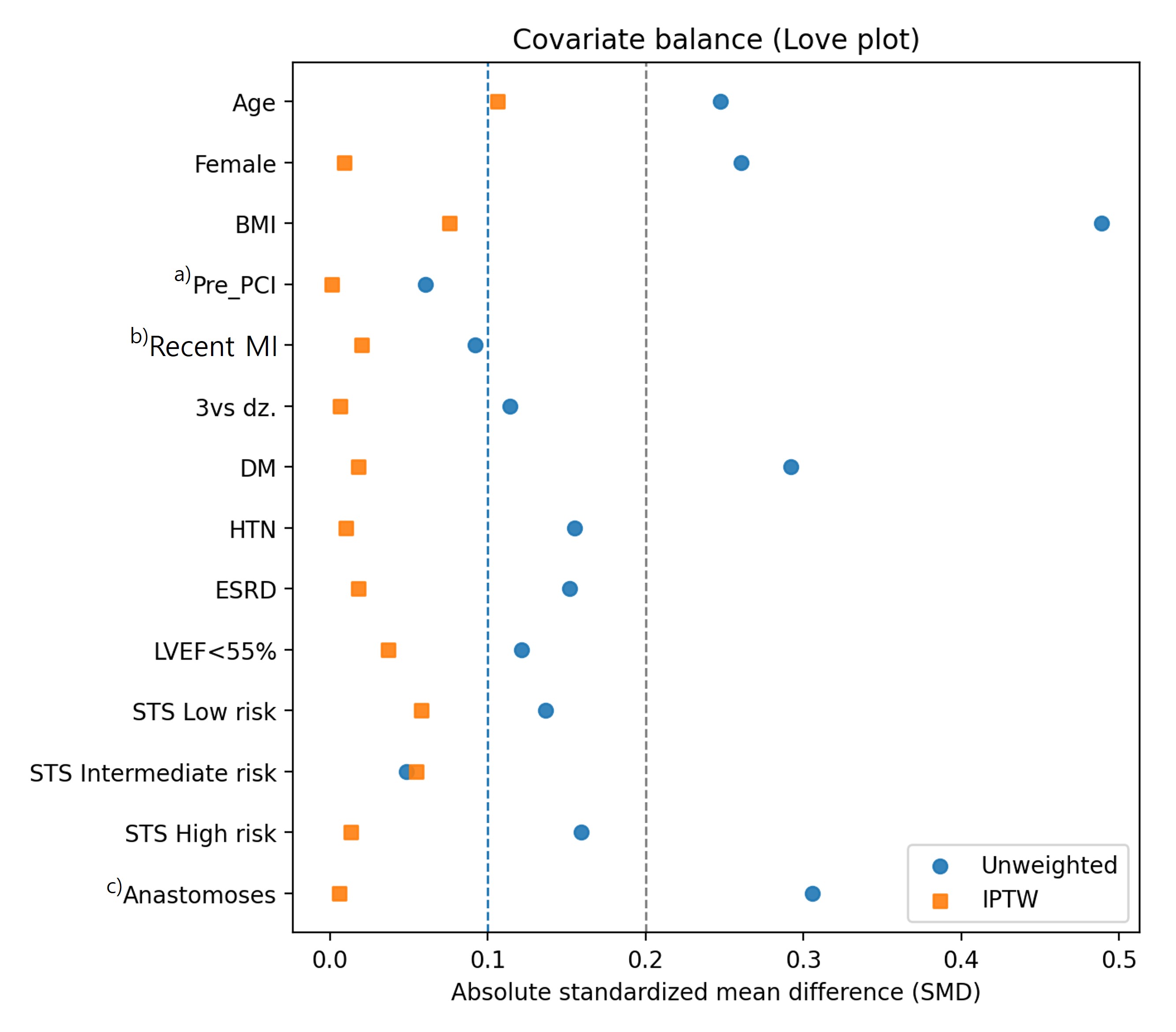

Supplement: Supplementary file 2 — Additional file 2: Figure E2. Covariate balance before and after IPTW (love plot). Love plot showing absolute standardized mean differences before (blue) and after (orange) inverse probability of treatment weighting for the LPP and no-LPP groups. Vertical reference lines indicate thresholds for excellent (0.10) and acceptable (0.20) balance. Abbreviations: BMI, body mass index; PCI, percutaneous coronary intervention; MI, myocardial infarction; 3vs dz., 3 vessel coronary artery disease; DM, diabetes mellitus; HTN, hypertension; ESRD, end-stage renal disease; LVEF, left ventricular ejection fraction; STS score, Society of Thoracic Surgeons score. a) History of previous percutaneous coronary intervention. b) Patients with acute myocardial infarction within 1 week prior to operation. c) Number of coronary anastomoses. [file 13019_2026_4241_MOESM2_ESM.jpg]
