# Supplementary material for: Association of left posterior pericardiotomy with postoperative atrial fibrillation in Isolated OPCAB: a propensity-weighted analysis
Source: J Cardiothorac Surg. 2026 May 2;21:437. doi: 10.1186/s13019-026-04241-3 (PMC13285440; doi:10.1186/s13019-026-04241-3)
Supplement: Supplementary file 3 — Additional file 3: Table E1. Characteristics of postoperative pericardial effusion in patients with and without left posterior pericardiotomy. Effusion size was graded according to the 2025 ACC concise guidance (small <1.0 cm, moderate 1.0–1.9 cm, large 2.0–2.5 cm, very large >2.5 cm). Of 283 patients, 15 (5.3%) had detectable PE; 13 in the no-LPP group and 2 in the LPP group. Abbreviations: LPP, left posterior pericardiotomy; POAF, postoperative atrial fibrillation [file 13019_2026_4241_MOESM3_ESM.docx]

**Table E1. Characteristics of postoperative pericardial effusion in patients with and without left posterior pericardiotomy.**

|  | **Number** | **Echocardiography (cm)** | **Pericardial effusion** | **Location** | **POAF** |
| --- | --- | --- | --- | --- | --- |
| **No-LPP** | 1 | 0.61 | Small | Posterior wall | X |
|  | 2 | 0.97 | Small | Right atrium | O |
|  | 3 | 0.77 | Small | Right atrium | O |
|  | 4 | 0.6 | Small | Posterior wall | X |
|  | 5 | 0.22 | Small | Posterior wall | X |
|  | 6 | 1.2 | Moderate | Right atrium | X |
|  | 7 | 0.62 | Small | Lateral wall | O |
|  | 8 | 0.4 | Small | Inferior wall | X |
|  | 9 | 0.43 | Small | Posterior wall | O |
|  | 10 | 1.19 | Moderate | Posterior wall | O |
|  | 11 | 0.34 | Small | Posterior wall | X |
|  | 12 | 1.48 | Moderate | Right atrium | X |
|  | 13 | 0.41 | Small | Right atrium | O |
| **LPP** | 14 | 0.91 | Small | Posterior wall | X |
|  | 15 | 1.66 | Moderate | Right atrium | X |
